# Concordance Between Trial Efficacy and Real-World Effectiveness of Sodium-Glucose Cotransporter 2 Inhibitors in Heart Failure With Reduced Ejection Fraction: A Systematic Review and Meta-Analysis

**DOI:** 10.7759/cureus.118487

**Published:** 2026-10-24

**Authors:** Ahmed Hassanien, Mohamed Elhenedy, Mohamed Elgohari

**Affiliations:** 1 Internal Medicine, East Lancashire Hospitals NHS Trust, Blackburn, GBR; 2 Cardiology, Blackpool Teaching Hospitals NHS Foundation Trust, Blackpool, GBR; 3 Internal Medicine, Al Haram Hospital, Cairo, EGY

**Keywords:** comparative effectiveness, heart failure with reduced ejection fraction, meta-analysis, real-world evidence, sglt2 inhibitors, systematic review

## Abstract

Randomized trials established the efficacy of sodium-glucose cotransporter 2 (SGLT2) inhibitors in heart failure with reduced ejection fraction (HFrEF). We assessed whether their effectiveness in routine practice is concordant with trial efficacy, appraising and pooling each evidence stream separately. PubMed/MEDLINE, Cochrane Central Register of Controlled Trials (CENTRAL), and ClinicalTrials.gov were searched from January 1, 2015 to July 2026 for randomized trials and comparative observational studies of SGLT2 inhibitors vs. placebo or nonuse in adults with left ventricular ejection fraction ≤40% reporting mortality or heart failure hospitalization. Risk of bias was assessed with the Cochrane Risk of Bias 2 and the Newcastle-Ottawa Scale. Adjusted all-cause mortality was pooled by random-effects meta-analysis with the Hartung-Knapp adjustment; other outcomes were synthesized narratively. Trial and observational estimates were never combined, and certainty was graded with the Grading of Recommendations Assessment, Development and Evaluation (GRADE) criteria. Eleven studies were included (two trials and nine observational). For all-cause mortality, the pooled observational HR (0.75, 95% CI 0.71-0.79; I² = 0%) indicated a larger apparent benefit than the pooled trial estimate (HR 0.87, 0.77-0.98). In one registry, the association attenuated to borderline nonsignificance after adjustment for background therapy. For heart failure hospitalization, a consistent trial benefit (HR 0.69, 0.62-0.78) contrasted with attenuated or null real-world estimates (HR 0.84-1.03). Real-world mortality associations with SGLT2 inhibitors in HFrEF are directionally concordant with trial efficacy, but their larger magnitude most plausibly reflects residual confounding, including co-prescription of other guideline-directed therapy. Real-world hospitalization estimates diverged from trials, likely reflecting outcome ascertainment. With very low-certainty observational evidence, the trial estimate remains the best guide to causal benefit.

## Introduction and background

Heart failure with reduced ejection fraction (HFrEF) carries a high burden of mortality and hospitalization despite modern therapy. Sodium-glucose cotransporter 2 (SGLT2) inhibitors have become a pillar of guideline-directed medical therapy (GDMT) on the strength of two landmark randomized controlled trials (RCTs), DAPA-HF (dapagliflozin) and EMPEROR-Reduced (empagliflozin), which demonstrated reductions in cardiovascular death and worsening heart failure [1,2]. A prespecified pooled analysis of both trials also showed a significant reduction in all-cause mortality [3].

In DAPA-HF, 4744 patients with New York Heart Association class II-IV heart failure and an ejection fraction of 40% or less were randomized to dapagliflozin 10 mg once daily or placebo in addition to recommended therapy; over a median of 18.2 months the primary composite of worsening heart failure or cardiovascular death occurred in 16.3% vs. 21.2% of patients (HR 0.74, 95% CI 0.65-0.85), with similar findings in patients with and without diabetes [1]. In EMPEROR-Reduced, 3730 comparable patients were randomized to empagliflozin 10 mg once daily or placebo; over a median of 16 months the primary composite of cardiovascular death or hospitalization for worsening heart failure occurred in 19.4% vs. 24.7% (HR 0.75, 95% CI 0.65-0.86), and the decline in estimated glomerular filtration rate was slower with empagliflozin [2]. Neither trial was powered individually to assess mortality. The prespecified meta-analysis of the two trials (8474 patients) estimated a 13% reduction in all-cause death, a 14% reduction in cardiovascular death and a 26% reduction in the composite of cardiovascular death or first hospitalization for heart failure, together with a lower risk of the composite renal endpoint (HR 0.62, 95% CI 0.43-0.90) [3].

Randomized trials establish efficacy under controlled conditions in selected populations. Whether these benefits are reproduced as effectiveness in the broader, older and more comorbid populations treated in routine practice is a distinct question, and one directly relevant to guideline confidence and clinical decision-making [4]. A growing body of observational research has examined SGLT2 inhibitor outcomes in real-world HFrEF cohorts, but this literature has largely described treatment uptake, or has blended randomized and observational estimates. Blending the two obscures the very gap between efficacy and effectiveness that matters to clinicians. It also leaves unexamined a key source of bias in observational HFrEF data: patients started on an SGLT2 inhibitor are more likely to be receiving the other guideline-directed therapies. To our knowledge, no previous review has appraised and pooled the randomized and observational evidence for SGLT2 inhibitors in HFrEF separately, assessed observational studies specifically for adjustment for background GDMT, and compared the two evidence streams outcome by outcome.

We therefore undertook a systematic review comparing outcomes with SGLT2 inhibitors in HFrEF between RCTs and real-world observational studies, appraising and pooling each evidence stream separately and then examining their concordance across all-cause mortality, cardiovascular mortality, heart failure hospitalization and their composite.

## Review

Methods

Protocol, Registration, and Ethics

The review was prospectively registered with PROSPERO (CRD420251077971) and is reported in accordance with the PRISMA 2020 statement [5]. Amendments to the registered protocol, covering the databases searched, the analytic method and the review team, were logged in July 2026 before completion of data synthesis; a third reviewer was added to the registered team in October 2026. Two departures from the registered protocol are noted: citation searching, reference list checking and contact with authors were planned but not undertaken, and screening and data extraction were performed by one reviewer and verified by a second rather than independently in duplicate. Ethical approval and informed consent were not required because the review used only previously published aggregate data.

Eligibility Criteria

Inclusion and exclusion criteria are presented in Table 1. In brief, we included studies of adults (≥18 years) with HFrEF (left ventricular ejection fraction (LVEF) ≤40%) receiving an SGLT2 inhibitor. For the randomized stream, the comparator was placebo; for the observational stream, nonusers of SGLT2 inhibitors. Eligible outcomes were all-cause mortality, cardiovascular mortality, heart failure hospitalization, or the composite of cardiovascular death or heart failure hospitalization. RCTs and comparative observational studies (cohort, registry, or propensity-matched designs) were eligible. Where a study defined HFrEF by diagnostic code without measured ejection fraction, this was prespecified as a source of indirectness and examined by sensitivity analysis excluding that study.

**Table 1:** Inclusion and exclusion criteria. HF, heart failure; HFrEF, heart failure with reduced ejection fraction; LVEF, left ventricular ejection fraction; RCT, randomized controlled trial; SGLT2, sodium-glucose cotransporter 2

Criterion	Definition
Population	Adults ≥18 years with HFrEF (LVEF ≤40%)
Intervention	SGLT2 inhibitor (any agent, any dose)
Comparator	Placebo (randomized trials) or nonuse/noninitiation (observational studies)
Outcomes	All-cause mortality, cardiovascular mortality, HF hospitalization, or composite of cardiovascular death or HF hospitalization
Study design	RCTs, observational cohort studies, registry studies, propensity-matched studies
Exclusion: comparator	Lack of nonuser/placebo comparator; head-to-head SGLT2 inhibitor comparisons
Exclusion: outcomes	No hard clinical outcome; only biomarkers or intermediate outcomes
Exclusion: population	Non-HFrEF populations; mixed HFrEF and non-HFrEF without separate HFrEF analysis
Exclusion: intervention	Bundled guideline-therapy interventions where SGLT2 inhibitor effect not isolated
Publication type	Peer-reviewed full-text publications only; no preprints or conference abstracts

Information Sources and Search Strategy

PubMed/MEDLINE, the Cochrane Central Register of Controlled Trials (CENTRAL), and ClinicalTrials.gov were searched for records published from January 1, 2015 to July 2026, without language restrictions. Search strategies are presented in Table 2. Search terms combined SGLT2 inhibitor terms, heart failure and HFrEF terms, and outcome terms, using both free-text and controlled vocabulary. Embase, Scopus and Web of Science were not searched, and citation searching was not performed; the search is therefore not exhaustive for observational evidence, which is acknowledged as a limitation. Preprints and conference abstracts were not eligible; only peer-reviewed full-text reports were included. Because CENTRAL and ClinicalTrials.gov index trials, they informed the randomized stream only; observational studies were identified from PubMed/MEDLINE.

**Table 2:** Search strategies by source. All sources were searched for records published from January 1, 2015 to July 2026.

Database	Search strategy
PubMed/MEDLINE	(SGLT2 inhibitor* OR sodium glucose cotransporter 2 OR empagliflozin OR dapagliflozin OR canagliflozin OR ertugliflozin) AND (heart failure OR HF OR HFrEF) AND (mortality OR death OR hospitalization OR effectiveness OR real-world OR observational)
Cochrane CENTRAL	#1 SGLT2 inhibitor* OR sodium glucose cotransporter 2 OR empagliflozin OR dapagliflozin OR canagliflozin OR ertugliflozin; #2 heart failure OR HF OR HFrEF; #3 mortality OR death OR hospitalization OR effectiveness OR real-world; #1 AND #2 AND #3
ClinicalTrials.gov	("SGLT2 inhibitor" OR dapagliflozin OR empagliflozin OR canagliflozin OR ertugliflozin OR sotagliflozin) AND ("HFrEF" OR "heart failure with reduced ejection fraction" OR "systolic heart failure") AND ("real-world" OR observational OR cohort OR registry OR RCT OR "randomized controlled trial") AND (mortality OR hospitalization OR death OR survival)

Selection Process and Data Extraction

Records were screened by title and abstract and then in full text, with a specific reason recorded against prespecified codes for every full-text exclusion. Data were extracted into a standardized form capturing population, exposure and comparator definitions, adjustment method and whether background GDMT was modelled, follow-up duration, and adjusted HRs for each outcome. Screening and extraction were performed by one reviewer (AH) and independently verified against the source publications by a second reviewer (ME); discrepancies were resolved by consensus.

Risk of Bias Assessment

Randomized trials were appraised with the Cochrane Risk of Bias 2 (RoB 2) tool [6] and observational studies with the Newcastle-Ottawa Scale (NOS) [7], with particular attention to comparability (specifically, adjustment for background GDMT) and to outcome ascertainment. NOS scores were converted to overall categories using a prespecified rule (7-9 stars, low risk; 5-6, moderate; <5, high). Assessments were made by one reviewer (AH) and verified by a second (ME).

Data Synthesis

Adjusted HRs were pooled where an outcome was reported with a consistent definition by at least three observational studies, using a random-effects model with restricted maximum-likelihood estimation of between-study variance and the Hartung-Knapp adjustment to the CI. The Hartung-Knapp adjustment was preferred over the DerSimonian-Laird approach because the latter tends to underestimate uncertainty when few studies are pooled [8]. SEs were derived from published CIs on the log-hazard-ratio scale [9]; where a reported interval was mildly asymmetric, the SE was approximated from the full interval width, and sensitivity analysis confirmed this had no material effect. Heterogeneity was quantified with the I² statistic and a 95% prediction interval was calculated. Robustness was assessed by leave-one-out analysis, by excluding the most heavily weighted study, and by adding one further adjusted cohort. The pooling condition was met only for all-cause mortality; other outcomes were synthesized narratively. Randomized and observational estimates were appraised and pooled separately and were never statistically combined; the prespecified pooled trial estimate is presented alongside the pooled observational estimate for comparison only. Analyses were performed in R (R Foundation for Statistical Computing, Vienna, Austria) using the meta package [10]. Certainty of evidence was assessed using the Grading of Recommendations Assessment, Development and Evaluation (GRADE) criteria [11].

Reporting Bias

Formal statistical assessment of small-study effects was not undertaken, as fewer than ten studies contributed to any pooled outcome, below the threshold at which funnel-plot asymmetry or Egger’s test can be reliably interpreted [12]. Completeness of the randomized evidence was instead assessed by cross-checking ClinicalTrials.gov for completed but unpublished eligible trials.

Results

Study Selection

Figure 1 shows the PRISMA 2020 flow diagram of study selection. In the real-world stream, 264 records were screened, 238 were excluded at title and abstract, and 26 reports were assessed in full text, of which 17 were excluded, leaving nine observational studies. In the randomized stream, 2168 records were identified (PubMed n = 435, Cochrane CENTRAL n = 1460, ClinicalTrials.gov n = 273); after removal of 942 CENTRAL records before screening, 1226 records were screened, and 1222 were excluded, leaving four records (two primary publications and two trial registrations) that corresponded to two eligible trials. No additional hard-outcome SGLT2 inhibitor trial in HFrEF was identified in any source.

**Figure 1:**
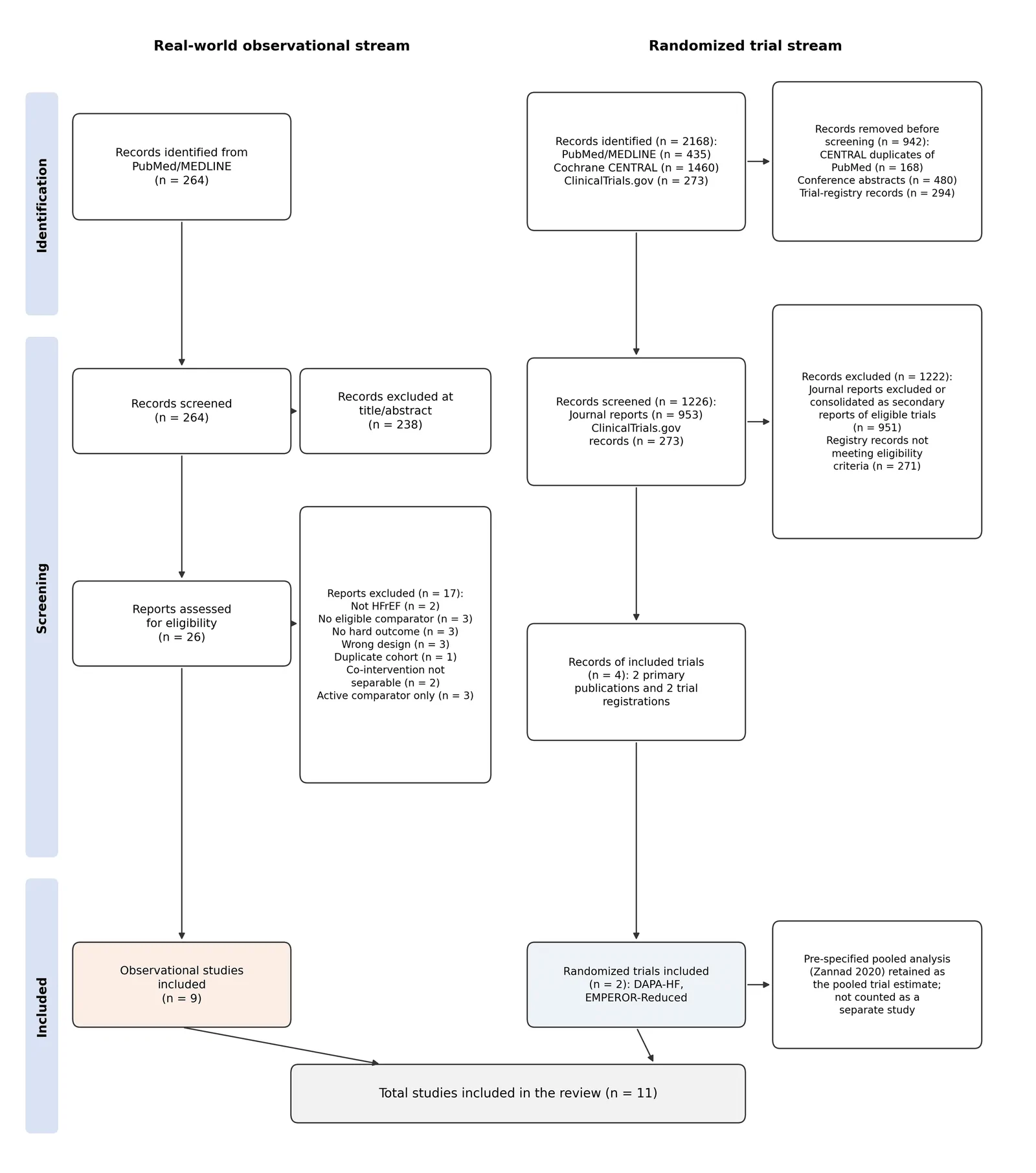
PRISMA flow diagram. CENTRAL, Cochrane Central Register of Controlled Trials; HFrEF, heart failure with reduced ejection fraction

Study Characteristics

Study characteristics are summarized in Table 3 [1,2,13-21]. The two trials randomized 8474 patients in total to dapagliflozin or empagliflozin vs. placebo, with median follow-up of 16-18 months. The nine observational studies (n = 150 to 70 042) comprised national registers, administrative and registry cohorts, an electronic health record network, a prospective multicenter hospital registry, and single-center or implementation cohorts. Comparators were nonusers or noninitiators of SGLT2 inhibitors. Five studies adjusted for background GDMT within the propensity or regression model; the remainder reported crude estimates only.

**Table 3:** Characteristics of included studies. The table lists the 11 included studies (two randomized trials and nine observational studies). The prespecified pooled analysis of the two trials provided the pooled trial estimate and is not counted as a separate study [3]. EHR, electronic health record; GDMT, guideline-directed medical therapy; IPTW, inverse-probability-of-treatment weighting; PSM, propensity-score matching; RCT, randomized controlled trial; SGLT2, sodium-glucose cotransporter 2

Study	Design	N (SGLT2 inhibitor/comparator)	Comparator	Follow-up	Adjusted for background GDMT
McMurray et al. (2019) [1], DAPA-HF	RCT	4744 (2373/2371)	Placebo	18.2 months	Randomized
Packer et al. (2020) [2], EMPEROR-Reduced	RCT	3730 (1863/1867)	Placebo	16 months	Randomized
Svanström et al. (2024) [13], Denmark	National registers, IPTW	21 462 (6776/14 686)	Nonuser	Median 0.8-1.1 years	Yes - all major classes
Brownell et al. (2026) [14], US Medicare	Registry, overlap weighting	8847 (1464/7383)	Eligible, not prescribed	12 months	Yes
Kobayashi et al. (2026) [15], TriNetX	EHR network, propensity matched	70 042 (14 670 pairs)	Not initiated ≤6 weeks	12 months	Yes (matched)
Leiner et al. (2026) [16], H2-registry	Prospective registry, Cox	810 (513/297)	No SGLT2 inhibitor at discharge	12 months	Yes
Balaguer Germán et al. (2024) [17], Spain	Single-center ≥75 years, Cox + PSM	364 (55/309)	Nonuser	33 months	Partial
Cetin et al. (2025) [18], ablation cohort	Retrospective cohort; mortality comparison unadjusted	246 (71/175)	Nonuser	347 days	No (groups balanced at baseline)
Towiargi et al. (2022) [19]	Retrospective, crude	195 (71/124)	Other antidiabetic	2020-21	No
Burgos et al. (2024) [20], Argentina	Implementation cohort, crude	237	Not initiated in hospital	90 days	No
Okoroike et al. (2023) [21], INSIGHT-HF, US	Implementation cohort, crude	150 (75/75)	Not initiated in hospital	30 days	No

Results of Individual Studies

The principal findings of the individual observational studies are summarized below, beginning with the five cohorts that reported adjusted estimates.

Svanström et al. used linked Danish national registers (July 2020 to June 2023) to compare 6776 patients aged 45 years or older with a LVEF of 40% or less who initiated an SGLT2 inhibitor (79% dapagliflozin, 21% empagliflozin) with 14 686 patients who continued other standard-of-care heart failure drugs [13]. The mean age of SGLT2 inhibitor users was 71.2 years, 70% were male, and 20% had type 2 diabetes. There were 374 deaths among users (5.8 per 100 person-years) and 1602 among nonusers (8.5 per 100 person-years), giving a weighted HR for all-cause mortality of 0.75 (95% CI 0.66-0.85), with consistent estimates in patients with and without diabetes [13].

Brownell et al. studied Medicare beneficiaries aged 65 years or older hospitalized for HFrEF in the Get With The Guidelines-Heart Failure registry between July 2021 and June 2023 [14]. Of 8847 eligible patients not prescribed an SGLT2 inhibitor at admission (median age 77 years, 40% women, median ejection fraction 28%), 1464 (16.5%) were initiated by discharge. After overlap weighting, initiation was associated with lower all-cause mortality (HR 0.76, 95% CI 0.67-0.86), all-cause readmission (HR 0.89, 95% CI 0.81-0.97) and heart failure readmission (HR 0.84, 95% CI 0.75-0.95) over 12 months, with consistent findings across subgroups defined by age, sex, diabetes, chronic kidney disease and ejection fraction [14].

Kobayashi et al. analyzed the TriNetX research network, identifying 70 042 patients with a first heart failure hospitalization between September 2021 and December 2023, of whom 21.3% were initiated on an SGLT2 inhibitor within six weeks [15]. In 14 670 propensity-matched pairs (mean age 64 years, 41.6% female), initiation was associated with lower one-year all-cause mortality (HR 0.75, 95% CI 0.69-0.83), all-cause hospitalization (HR 0.86, 95% CI 0.83-0.91) and emergency department visits (HR 0.91, 95% CI 0.86-0.96) [15].

Leiner et al. reported 810 patients with HFrEF from the prospective German H2-registry (513 treated with an SGLT2 inhibitor at discharge and 297 not treated; median age 70 years, 23% female, median ejection fraction 30%, 39% with type 2 diabetes) [16]. Renin-angiotensin-aldosterone system inhibitors, beta-blockers, and mineralocorticoid receptor antagonists were more commonly used in the SGLT2 inhibitor group. All-cause mortality was lower with SGLT2 inhibitor treatment in unadjusted analysis (HR 0.53, 95% CI 0.33-0.88), but in multivariable analysis only mineralocorticoid receptor antagonist treatment was independently associated with lower mortality (HR 0.41, 95% CI 0.23-0.74); among patients treated continuously with an SGLT2 inhibitor during follow-up, the adjusted association remained significant (HR 0.54, 95% CI 0.30-0.99) [16].

Balaguer Germán et al. enrolled 364 consecutive patients aged 75 years or older with HFrEF at a single center (mean age 84.1 years, mean ejection fraction 29.8%), of whom 55 received an SGLT2 inhibitor [17]. Over a median follow-up of 33 months, there were 122 deaths; SGLT2 inhibitor treatment was associated with lower all-cause mortality in multivariable Cox regression (HR 0.39, 95% CI 0.19-0.82) and in a propensity-score-matched analysis (HR 0.39, 95% CI 0.16-0.97) [17].

The remaining four studies reported unadjusted comparisons. Cetin et al. studied 246 patients with HFrEF undergoing cryoballoon ablation for atrial fibrillation (71 SGLT2 inhibitor users and 175 nonusers; median follow-up 347 days), in whom mortality (9.9% vs. 14.9%) and heart failure hospitalization (16.9% vs. 23.4%) were secondary endpoints and were lower among users [18]. Towiargi et al. compared 71 patients with type 2 diabetes and HFrEF receiving empagliflozin with 124 receiving other antidiabetic agents and found no significant difference in cardiovascular death or hospital admission, with no deaths in either group [19]. Burgos et al. reported 237 patients hospitalized with acute heart failure and an ejection fraction below 40% (mean age 76 years); the 90-day composite of all-cause death, heart failure rehospitalization, or unplanned heart failure visit was less frequent with in-hospital initiation (23.9% vs. 44.4%) [20]. In the INSIGHT-HF study, 30-day heart failure readmission was lower with inpatient initiation (9.3% vs. 22.7%), whereas cardiovascular mortality did not differ significantly (4% vs. 10.7%) [21].

Risk of Bias

Both trials were judged at low risk of bias across all five RoB 2 domains. Among the observational studies, the Danish national-register [13] and US Medicare [14] studies scored highest on the NOS (9/9, low risk), with extensive propensity-based adjustment including all major guideline-therapy classes, objective linkage-based outcome ascertainment and near-complete follow-up; the German H2-registry [16] also scored in the low-risk range (8/9) despite some loss to follow-up. The TriNetX study [15], despite its size, scored lower (5/9, moderate), with concerns that in our judgment approach the moderate-to-high range: HFrEF was defined by diagnostic code rather than measured ejection fraction, and network electronic health record data are prone to incomplete outcome capture. The very elderly single-center study [17] scored 7/9. The remaining four cohorts reported crude estimates only; they fell below the comparability threshold, were classified as high risk without full item-level scoring, and contributed to narrative synthesis alone (Table 4).

**Table 4:** Risk of bias summary. NOS scores were converted to overall categories using a prespecified rule (7-9 stars, low risk; 5-6, moderate; <5, high). GDMT, guideline-directed medical therapy; NOS, Newcastle-Ottawa Scale; RoB 2, Cochrane Risk of Bias 2

Study	Tool	Comparability/GDMT adjustment	Overall judgment
McMurray et al. (2019) [1], DAPA-HF; Packer et al. (2020) [2], EMPEROR-Reduced	RoB 2	Randomized (balanced by design)	Low
Svanström et al. (2024) [13], Denmark	NOS	All GDMT classes in propensity model	Low
Brownell et al. (2026) [14], US Medicare	NOS	GDMT in propensity model	Low
Kobayashi et al. (2026) [15], TriNetX	NOS	GDMT matched; code-based population (NOS 5)	Moderate
Leiner et al. (2026) [16], H2-registry	NOS	GDMT in Cox model; some loss to follow-up (one star deducted)	Low
Balaguer Germán et al. (2024) [17], Spain	NOS	Partial GDMT adjustment; single-center	Low
Cetin et al. (2025) [18], ablation cohort; Towiargi et al. (2022) [19]; Burgos et al. (2024) [20], Argentina; Okoroike et al. (2023) [21], INSIGHT-HF, US)	NOS	Crude estimates only	High

Synthesis of Outcomes

Quantitative pooling was possible only for all-cause mortality; all other outcomes were synthesized narratively. Certainty of evidence for each outcome is summarized in Table 5.

**Table 5:** Certainty of evidence (GRADE). Randomized evidence begins at high certainty and observational evidence at low certainty; observational outcomes were downgraded to very low for serious risk of bias (residual confounding), and trial mortality outcomes were downgraded once for inconsistency between the two trials. No outcome was upgraded. Imprecision was not rated reassuring for the pooled real-world mortality estimate despite the narrow CI, because the dominant limitation is residual confounding rather than random error. * Differing composite definitions (cardiovascular vs. all-cause death) between real-world studies. GRADE, Grading of Recommendations Assessment, Development and Evaluation; HF, heart failure; HHF, hospitalization for heart failure

Outcome	Evidence stream	Effect (HR, 95% CI)	Certainty
All-cause mortality	Trials (pooled)	0.87 (0.77-0.98)	Moderate
	Real-world (pooled)	0.75 (0.71-0.79)	Very low
Cardiovascular mortality	Trials (pooled)	0.86 (0.76-0.98)	Moderate
	Real-world (single study)	0.77 (0.64-0.92)	Very low
HF hospitalization	Trials (pooled)	0.69 (0.62-0.78)	High
	Real-world	0.84 to 1.03	Very low
Composite cardiovascular death/HHF	Trials (pooled)	0.74 (0.68-0.82)	High
	Real-world	0.94/0.80*	Very low

All-cause mortality: In the trials, dapagliflozin reduced all-cause mortality (HR 0.83, 95% CI 0.71-0.97) whereas empagliflozin did not reach significance (HR 0.92, 0.77-1.10); the pooled trial estimate was 0.87 (0.77-0.98) [1-3]. Pooling the four large, general population observational cohorts [13-16] gave a summary HR of 0.75 (95% CI 0.71-0.79), with no detectable heterogeneity (I² = 0%; 95% prediction interval 0.68-0.83) (Figure 2). The estimate was robust: leave-one-out analysis did not materially change it; excluding the most heavily weighted study (TriNetX, 47% weight) left it essentially unchanged; and adding a very elderly single-center cohort [17] (HR 0.39, 0.16-0.97) altered it only marginally (0.75, 0.70-0.80). The I² point estimate was 0%, but its CI was wide, reflecting the small number of pooled studies rather than established homogeneity. In the German registry, the crude association (HR 0.53) attenuated to borderline nonsignificance after multivariable adjustment (HR 0.60, 0.35-1.00), while mineralocorticoid receptor antagonist use remained independently associated with survival (HR 0.41, 0.23-0.74) [16].

**Figure 2:**
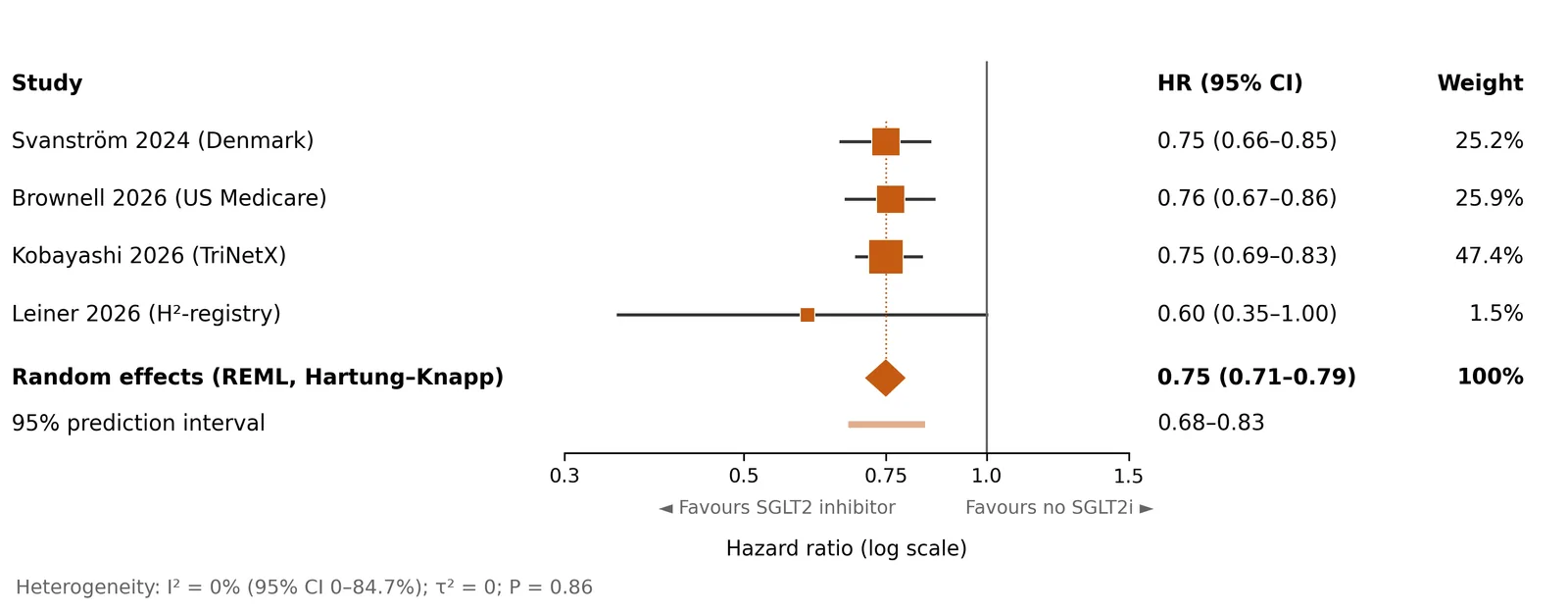
Forest plot of adjusted all-cause mortality in the observational cohorts. Random-effects meta-analysis (restricted maximum likelihood with Hartung-Knapp adjustment) of the four large general population observational cohorts: Svanström et al. [13], Brownell et al. [14], Kobayashi et al. [15], and Leiner et al. [16]. Squares show study-reported adjusted HRs, sized by weight; the diamond shows the pooled estimate (HR 0.75, 95% CI 0.71-0.79) and the bar shows the 95% prediction interval. The pooled trial estimate (HR 0.87, 0.77-0.98) is shown for comparison only and was never statistically combined with these observational data [3]. REML, restricted maximum likelihood

Cardiovascular mortality: Trial estimates were concordant with all-cause mortality (DAPA-HF 0.82, 0.69-0.98; EMPEROR-Reduced 0.92, 0.75-1.12; pooled 0.86, 0.76-0.98) [1-3]. Only one observational study, the Danish national-register analysis [13], reported an adjusted cardiovascular-mortality estimate (0.77, 0.64-0.92), closely concordant with the pooled trial figure; the comparison therefore rests on a single data point.

Heart failure hospitalization: Both trials showed a consistent reduction, with a pooled first-hospitalization estimate of 0.69 (0.62-0.78) [3]. Real-world estimates were attenuated or null: heart failure readmission 0.84 (0.75-0.95) in the Medicare cohort [14] and 1.03 (0.92-1.15) in the Danish registers [13]. The TriNetX study [15] reported only all-cause hospitalization (0.86, 0.83-0.91), and the German registry [16] could not undertake a time-to-event hospitalization analysis because exact event dates were not captured. The contrast between a pooled trial reduction of approximately 31% and real-world estimates ranging from a 16% reduction to no effect is most consistent with differences in outcome ascertainment (administrative coding vs. blinded adjudication, all-cause vs. heart failure-specific capture, and inconsistent handling of competing risks) rather than a true absence of benefit. The Danish investigators attributed their null result to routine coding practice, reporting a positive predictive value of 76% for heart failure hospitalization coding [13].

Composite of cardiovascular death or heart failure hospitalization: Trial composites were closely aligned (pooled 0.74, 0.68-0.82) [3]. Real-world composites were not directly comparable owing to differing definitions: the Danish composite, which matched the trial endpoint, was null (0.94, 0.85-1.04), tracking its null hospitalization component [13], whereas the Medicare composite incorporated all-cause rather than cardiovascular death (0.80, 0.73-0.88) [14]. Composite estimates were therefore not pooled. Trial and real-world estimates for each outcome are shown side by side in Figure 3.

**Figure 3:**
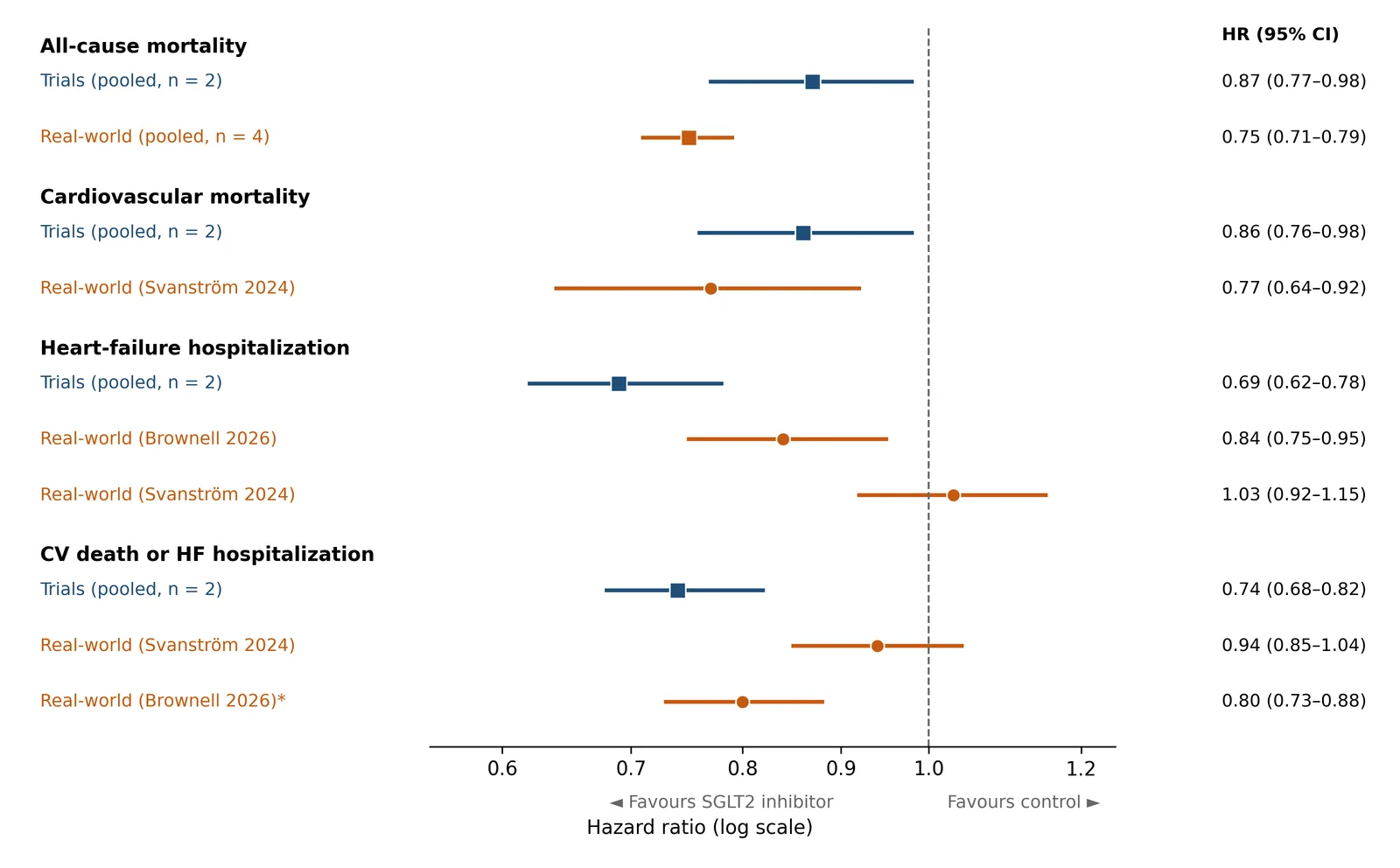
Trial efficacy vs. real-world effectiveness by outcome. Squares denote pooled estimates and circles denote individual real-world studies. Pooled trial estimates are from the prespecified meta-analysis [3] of DAPA-HF [1] and EMPEROR-Reduced [2]; the pooled real-world mortality estimate combines Svanström et al. [13], Brownell et al. [14], Kobayashi et al. [15], and Leiner et al. [16]; individual real-world estimates are from Svanström et al. [13] and Brownell et al. [14]. Trial and observational estimates are shown side by side for comparison only and were never statistically combined. The Brownell heart failure hospitalization estimate represents heart failure readmission. CV, cardiovascular; HF, heart failure; HR, hazard ratio; SGLT2, sodium-glucose cotransporter 2

Safety and Other Outcomes

Safety and treatment discontinuation were prespecified outcomes but were reported too sparsely and heterogeneously across the observational studies to permit synthesis. Descriptively, pooled trial data showed no excess over placebo in serious adverse events, volume depletion, severe hypoglycemia, fracture, lower-limb amputation or ketoacidosis [3]; in the few real-world studies reporting safety, discontinuation ranged from approximately 5% to 11%, driven mainly by urinary tract infection, hypotension and declining renal function. Other prespecified secondary outcomes (New York Heart Association class, quality of life, renal function, adherence and healthcare utilization) were too sparsely reported to synthesize.

Discussion

This review compared the efficacy of SGLT2 inhibitors in HFrEF, as established in randomized trials, with their effectiveness in routine clinical practice, appraising and pooling each evidence stream separately. Three findings emerged. First, for all-cause mortality, the two streams were concordant in direction but not in magnitude: the pooled observational estimate (0.75, 0.71-0.79) indicated a larger apparent protective association than the pooled trial estimate (0.87, 0.77-0.98). Second, this excess is most plausibly attributable to residual confounding, a conclusion supported from within the dataset itself. Third, for heart failure hospitalization, the streams diverged, a discordance better explained by differences in outcome ascertainment than by a true loss of treatment effect.

Comparison With Existing Evidence

The efficacy benchmark for this comparison is the prespecified pooled analysis of DAPA-HF and EMPEROR-Reduced [3]. Previous observational work has largely characterized treatment uptake, or has blended randomized and observational estimates, rather than contrasting them in an HFrEF-specific manner. Separating the two streams is the methodological contribution of the present review, since pooling efficacy and effectiveness into a single estimate obscures precisely the efficacy-effectiveness gap that is of interest. Our central observation, that the observational mortality estimate exceeds the randomized one, is consistent with meta-epidemiological evidence that mortality effects estimated from routinely collected data tend to be more favorable than those of subsequent randomized trials [22], and with the well-described tendency for treated patients to be healthier or better managed than comparators [23]. It should not be interpreted as observational data confirming or strengthening the trial result; a real-world estimate that exceeds the randomized benchmark is a signal of residual bias rather than of a larger true effect.

Why Real-World Mortality Effects Exceed Trial Estimates

Several mechanisms plausibly act in the same direction. Patients initiated on and persisting with a newer therapy tend to be more adherent and healthier overall, and initiation is less likely in the frailest patients [23]. Most importantly, SGLT2 inhibitor users in these cohorts were more likely to be receiving the remainder of GDMT: baseline data from the national register and registry studies show higher background use of renin-angiotensin inhibitors, beta-blockers and mineralocorticoid receptor antagonists among SGLT2 inhibitor users. Part of the apparent SGLT2 inhibitor effect in observational data is therefore attributable to the accompanying therapy. The German registry makes this concrete: once background therapy was modelled, the SGLT2 inhibitor association attenuated to nonsignificance and mineralocorticoid receptor antagonist use remained independently associated with survival [16]. Critically, the absence of statistical heterogeneity across the pooled cohorts does not argue against this interpretation. Heterogeneity statistics detect disagreement between studies; they cannot detect bias shared across studies [24]. If these cohorts share a common confounding structure, as is likely given similar routine-care populations and prescribing patterns, they will produce consistent and consistently overstated estimates. The pooled figure of 0.75 is therefore best understood as a reliable estimate of the observed association in routine care, not necessarily of the causal drug effect.

The Hospitalization Discordance

Trial hospitalization endpoints are captured by blinded, protocol-driven adjudication, whereas routine data rely on administrative discharge coding, which is less sensitive and specific. Real-world studies also varied in whether they counted heart failure-specific or all-cause admissions, first or recurrent events, and whether death was handled as a competing risk; one registry could not perform a time-to-event analysis at all. These factors limit direct comparability with trials, though the direction of any net bias cannot be established here. The divergence should therefore not be interpreted as evidence that SGLT2 inhibitors fail to prevent hospitalization in practice.

Clinical Implications

The findings are consistent with a mortality benefit of SGLT2 inhibitors in unselected HFrEF populations treated in routine care, supporting current guideline recommendations from the American Heart Association/American College of Cardiology/Heart Failure Society of America and the European Society of Cardiology [25-27]. These guidelines recommend SGLT2 inhibitors as one of the four foundational therapies for HFrEF, alongside a renin-angiotensin system inhibitor (preferably an angiotensin receptor-neprilysin inhibitor), a beta-blocker, and a mineralocorticoid receptor antagonist. The trial estimate remains the most reliable guide to causal benefit, and the larger real-world mortality estimates should not be interpreted as evidence of greater efficacy; rather, they reflect residual confounding from co-medication. The finding that mineralocorticoid receptor antagonist use remained independently associated with survival, even as the SGLT2 inhibitor estimate attenuated in adjusted analyses, underscores a critical principle: outcomes in HFrEF depend on the completeness of GDMT rather than any single agent [16]. Clinicians should ensure that eligible patients with HFrEF receive all four pillar therapies at tolerated doses before ascribing treatment responses to any individual component.

Strengths and Limitations

The review addressed a specific, clinically meaningful question with a prospectively registered protocol, a separate-stream design, searches across two bibliographic databases and a trial register, explicit exclusion reasons, and data extraction and risk of bias appraisal independently verified against the source publications by a second reviewer. The pooled mortality estimate was statistically homogeneous, robust to leave-one-out and sensitivity analyses, and used a Hartung-Knapp adjustment appropriate to the small number of studies [8].

Several limitations apply. The real-world evidence base is small and methodologically heterogeneous, and several contributing studies were at moderate-to-high risk of bias; the largest observational study defined HFrEF by diagnostic code rather than measured ejection fraction, although the pooled estimate did not depend on it. Embase, Scopus, and Web of Science were not searched, and citation searching was not performed, so the search is not exhaustive and additional eligible observational studies may exist. Screening and data extraction were performed by one reviewer with verification by a second, rather than independently in duplicate, which may have increased the risk of selection or extraction error. Quantitative pooling was possible only for all-cause mortality, and with few pooled studies, the CI, though Hartung-Knapp adjusted, rests on limited data. Exposure was defined at drug-class level in most observational studies, so agent- and dose-specific effects could not be examined. Certainty of evidence for all observational outcomes was very low by GRADE, driven by residual confounding. Finally, the interpretive weight of the review rests on comparing estimates from fundamentally different designs, which supports judgments about direction and concordance but not formal statistical equivalence.

Recommendations

For clinical practice: Current guideline recommendations to use SGLT2 inhibitors as standard-of-care GDMT in HFrEF are supported by both trial efficacy data and real-world effectiveness findings. Clinicians should prioritize ensuring all four GDMT pillars (renin-angiotensin system inhibitor or angiotensin receptor-neprilysin inhibitor, beta-blocker, mineralocorticoid receptor antagonist, and SGLT2 inhibitor) are at tolerated doses in every eligible patient, rather than focusing on maximizing individual agents. When prescribing SGLT2 inhibitors in routine practice, clinicians should monitor for euglycemic diabetic ketoacidosis (rare but serious), volume status (particularly in conjunction with diuretics), renal function, and genital mycotic infections [25,26].

For guideline developers and professional societies: The external validity of SGLT2 inhibitor efficacy in routine HFrEF populations is supported, but certainty remains very low. Guidelines should emphasize that real-world mortality estimates are likely overstated due to confounding from co-prescription of other life- saving therapies, and should reinforce that complete GDMT (not any single agent) drives outcomes. Future guideline updates should stratify recommendations by patient characteristics (age, renal function, type 2 diabetes status, ejection fraction tier) based on emerging real-world data.

For regulatory and health-policy stakeholders: Real-world mortality associations in broader, older and more comorbid populations than those enrolled in the pivotal trials were directionally consistent with trial efficacy, but safety outcomes were reported too sparsely in the observational studies to permit synthesis. Policy should therefore facilitate active surveillance and registry-based monitoring of SGLT2 inhibitor safety signals (volume depletion, renal decline, ketoacidosis, fracture) in routine use, and should support efforts to improve real-world GDMT uptake in underrepresented populations.

Future Directions

Higher-quality real-world evidence is needed to resolve key uncertainties. Priority areas include (1) study design: active-comparator, new-user cohort studies and target-trial emulation designs to reduce confounding compared to crude observational estimates [28]; (2) GDMT adjustment: explicit and complete model-based adjustment for all guideline-directed therapies (renin-angiotensin system inhibitor or angiotensin receptor-neprilysin inhibitor class and dose, beta-blocker type and dose, mineralocorticoid receptor antagonist use and loop-diuretic intensity), as incomplete adjustment is the dominant source of bias in current literature; (3) hospitalization outcomes: heart failure-specific, adjudicated hospitalization events analyzed by competing-risk methods, rather than administrative discharge coding; (4) standardization: uniform outcome definitions (e.g., time to first HF hospitalization vs. recurrent events, HF- specific vs. all-cause admissions, handling of death as competing event) to permit meta-analytic pooling; (5) agent and dose specificity: separate analysis of dapagliflozin vs. empagliflozin and dose-response effects; (6) population substratification: dedicated analyses in older adults, women and ethnic minority groups, who were underrepresented in the pivotal trials; (7) mechanistic understanding: investigation of whether HF hospitalization divergence reflects ascertainment differences alone, or partial loss of benefit in routine care.

International collaboration through established HF registries (for example, the ESC Heart Failure Long-Term Registry, Get With The Guidelines-Heart Failure and the National Heart Failure Audit in England and Wales) could accelerate the generation of higher-certainty real-world evidence without de novo patient recruitment. Investment in interoperable electronic health record systems and standardized outcome definitions would substantially improve future comparative-effectiveness research in HFrEF.

## Conclusions

In HFrEF, real-world mortality associations with SGLT2 inhibitors were directionally consistent with trial efficacy, but agreement was outcome-dependent, and the certainty of the observational evidence was very low. For all-cause mortality, the pooled observational effect (0.75, 0.71-0.79) exceeded the trial estimate (0.87, 0.77-0.98); this difference is most plausibly explained by residual confounding from co-prescription of other guideline therapies and patient selection bias, not by a greater true treatment effect. For heart failure hospitalization, the evidence streams diverged markedly (trial HR 0.69 vs. real-world HR 0.84-1.03), a pattern more consistent with differences in outcome ascertainment and adjudication than with loss of clinical efficacy in practice. These findings support the external validity of SGLT2 inhibitor efficacy in routinely treated HFrEF populations and reinforce guideline recommendations, while cautioning that observational mortality effects in administrative databases are likely substantially overstated. Given the small evidence base (nine observational studies) and very low certainty by the GRADE criteria, these conclusions remain provisional. Systematic implementation of higher-quality real-world evidence generation, with explicit GDMT adjustment, standardized outcome definitions, and diverse populations, is needed to establish whether trial benefits translate to broad-population effectiveness.
